# Hydrogel-assisted neuroregeneration approaches towards brain injury therapy: A state-of-the-art review

**DOI:** 10.1016/j.csbj.2018.10.011

**Published:** 2018-11-02

**Authors:** Vladimir A. Kornev, Ekaterina A. Grebenik, Anna B. Solovieva, Ruslan I. Dmitriev, Peter S. Timashev

**Affiliations:** a Institute for Regenerative Medicine, Sechenov University, 8-2 Trubetskaya st., Moscow 119991, Russian Federation; b N. N. Semenov Institute of Chemical Physics, Russian Academy of Sciences, 4 Kosygina st., Moscow 117977, Russian Federation; c School of Biochemistry and Cell Biology, University College Cork, Cork, Ireland; d Institute of Photonic Technologies, Research Center “Crystallography and Photonics” Russian Academy of Sciences, 2 Pionerskaya st., Troitsk, Moscow 108840, Russian Federation

**Keywords:** biomaterials, brain injury, hydrogel, nerve tissue engineering, neural stem cells, stroke

## Abstract

Recent years have witnessed the development of an enormous variety of hydrogel-based systems for neuroregeneration. Formed from hydrophilic polymers and comprised of up to 90% of water, these three-dimensional networks are promising tools for brain tissue regeneration. They can assist structural and functional restoration of damaged tissues by providing mechanical support and navigating cell fate. Hydrogels also show the potential for brain injury therapy due to their broadly tunable physical, chemical, and biological properties. Hydrogel polymers, which have been extensively implemented in recent brain injury repair studies, include hyaluronic acid, collagen type I, alginate, chitosan, methylcellulose, Matrigel, fibrin, gellan gum, self-assembling peptides and proteins, poly(ethylene glycol), methacrylates, and methacrylamides. When viewed as tools for neuroregeneration, hydrogels can be divided into: (1) hydrogels suitable for brain injury therapy, (2) hydrogels that do not meet basic therapeutic requirements and (3) promising hydrogels which meet the criteria for further investigations. Our analysis shows that fibrin, collagen I and self-assembling peptide-based hydrogels display very attractive properties for neuroregeneration.

## Graphical abstract

**Figure:**
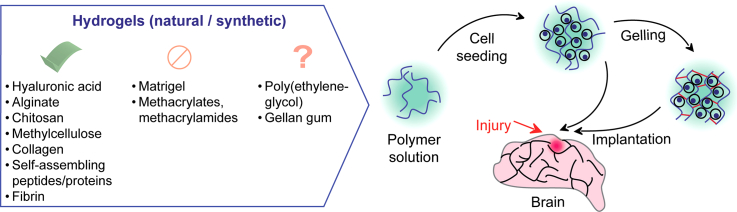

## 1 Introduction

The nervous system is essential for performing motor, sensory, and autonomic functions including somatic injury repair. However, the nervous system as a whole and the central nervous system (CNS) in particular have limited capacity for regeneration, which makes the effects of neurotrauma, ischemia, hemorrhage, or neurodegenerative disease devastating and often irreversible [1]. In part, it is caused by the intrinsic properties of neural parenchyma (insufficiency of progenitor neural cells in the adult nervous system and a slow ability of mature neural cells to regenerate, proliferate, and migrate [2]) and the microenvironment formed as a result of injury. This can lead to destruction of the blood-brain barrier (BBB), cytotoxicity (due to release of proteases, free radicals and other compounds from necrotic cells), trophic and oxygen deprivation. In the CNS, such a hostile microenvironment can only promote partial self-regeneration resulting in the formation of glial scars and cysts [[3], [4], [5]].

At present, there is no effective clinically applicable treatment for functional recovery after CNS injury, as new therapeutic strategies for enhancing endogenous repair mechanisms of the nervous system become ineffective. Mainly, this is due to the short half-life and systemic effects of injectable growth factors and the poor survival, differentiation, and migration of transplanted stem cells. One of the most promising approaches to address these issues is associated with the use of hydrogels, the three-dimensional (3D) networks formed by hydrophilic polymers containing up to 90% (w/w) of water [6]. Hydrogels possess tunable physical and chemical properties [[7], [8], [9]], which in the context of brain regeneration, are expected to meet the following requirements:

I. Injectability, shear-thinning and self-healing (thixotropy). The ability of hydrogel to support both viscous flow under shear stress (shear-thinning during injection) and time-dependent recovery upon relaxation (self-healing after injection at the injury site) are the major requirements for minimally invasive surgery [10,11].
II. Biocompatibility, low cytotoxicity and immunogenicity and the lack of mutagenicity. Hydrogel should not be physically separated from the host tissue by an inflammation-induced avascular glial scar and it should not harm the graft or host cells [12,13].
III. Biodegradability. Ideally, this should be coordinated and fine-tuned by the growing host tissue and lead to complete disappearance over time [12,13].
IV. Interactivity. The hydrogel-forming polymer should have adhesive molecular sequences capable of promoting graft and host cell proliferation, migration, and differentiation [12,13].
V. Porosity. The pores of optimal size and interconnectivity will facilitate spatial cell distribution, migration, extracellular matrix formation, and nutrient and oxygen exchange [7,12].
VI. Lack of swelling. This is important for preventing tissue compression and subsequent damage [14].

Hydrogels are of great interest for future clinical applications in neuroregeneration as they deal with both mentioned problems (insufficiency of progenitor cells and injury-induced microenvironment) [6,7]: they can serve as local transport systems for the delivery of drugs and signaling molecules directly to the injury site, and as scaffolds providing suitable physical support, substrates for adhesion, optimal nutrient and oxygen exchange, and protection to host and graft cells, thereby facilitating extracellular matrix formation. The main role of hydrogels in neuroregeneration is to exert control over the host neural tissue and grafted cell fate by supporting attachment, neurite outgrowth, proliferation, migration, differentiation, and viability [15].

**Fig. 1:**
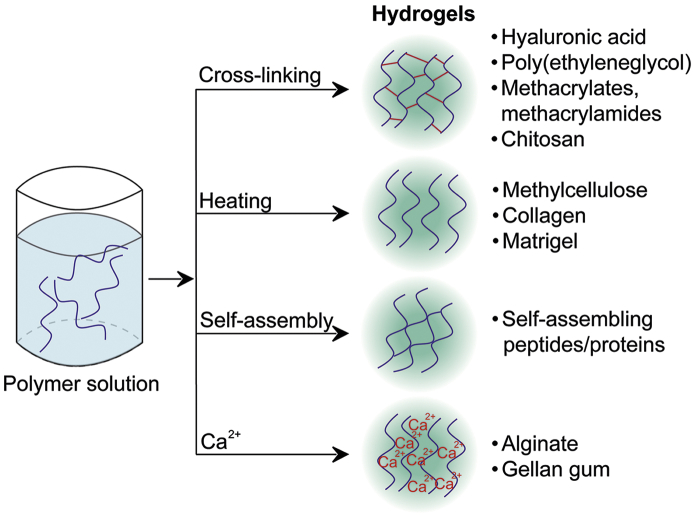
Schematic representation of different hydrogels and their formation mechanisms.

While hydrogel types can be divided and have been classified based on a variety of their properties in the literature [9,[15], [16], [17], [18], [19], [20], [21], [22]], our review focuses on the source-chemical classification and the formation mechanisms, with results provided from both *in vitro* and *in vivo* studies (Fig. 1).

## 2 Natural polymers

### 2.1 Hyaluronic acid

Hyaluronic acid (HA) is a linear polysaccharide that consists of two alternating units, β-1,4-D-glucuronic acid and β-1,3-N-acetyl-D-glucosamine. It is one of the most basic and physiologically relevant extracellular matrix components discovered [23,24]. HA is ubiquitous in the CNS where it is dispersed in the neuropil and forms perineuronal nets (PNNs) [25]. HA has been extensively studied in terms of neuroregeneration (reviewed in [10,15,17,19,21,[26], [27], [28], [29], [30], [31]]).

#### 2.1.1 In vitro studies

In 2009, Pan and co-workers tested EDC (1-Ethyl-N,N-dimethylaminopropyl carbodiimide)-cross-linked HA hydrogels for supporting rat embryonic NPCs (neural progenitor cells) viability and differentiation [32]. The group sought to determine the effects of HA modifications with either an antibody against the Nogo receptor or poly-L-lysine (PLL). They found that HA supported the viability of NPC independently on modification due to its cavernous structure providing sufficient space and nutrient supply. HA preferentially directed NPC growth toward neurons but it was non-adhesive for this cell line without the modifications. Similar results were found in the study on rat embryonic neural stem cells (NSC) by Ren et al. [33] where HA-PLL did not support NSC differentiation towards oligodendrocytes. These studies are in agreement with the 2009 *in vitro* study on EDC-cross-linked HA hydrogel, where Wei and co-workers examined primary rat hippocampal neurons for viability and differentiation in unmodified, modified with Nogo receptor antibody and PLL-matrices[34]. The results confirmed that HA alone was unable to stimulate differentiation. In 2009, Nakaji-Hirabayashi et al. investigated the effects of EDC-cross-linked HA hydrogel modified with BDNF (brain-derived neurotrophic factor) with rat fetal NSCs [35]. This study also confirmed comparative insufficiency of HA to support cell viability.

In 2010, Seidlits et al. produced methacrylate-cross-linked HA (HAMA) hydrogel to study neural cell growth. [23]. The hydrogel’s bulk compressive modulus varied depending on its metacrylate (MA) content, while the softest hydrogel (lowest MA:HA ratio) demonstrated the most efficient mouse embryonic NSCs differentiation. Similar results were obtained by Hachet et al. [36], who demonstrated that cell adhesion to HA was mediated by CD44 and RHAMM (receptor for hyaluronic acid-mediated motility). However, the authors used non-neural mouse embryo fibroblast NIH 3T3 and human cervical carcinoma HeLa cell lines.

In 2009, Wang et al. constructed a delivery system based on EDC-cross-linked HA, which also embedded BDNF and VEGF (vascular endothelial growth factor)-loaded PLGA (poly[lactic-co-glycolic acid]) microspheres [37]. Such scaffolds provided stable release of both BDNF and VEGF but were not capable of supporting survival and proliferation of rat embryonic NSCs.

In 2014, McMurtrey reported on PEG (poly[ethylene glycol]) diacrylate-cross-linked HA hydrogel modified with polycaprolactone (PCL) nanofibers, PCL nanofibers mixed with gelatin, and PCL with laminin coating and their effects on SH-SY5Y human neuroblastoma cells [38]. The paper also confirmed the poor ability of unmodified HA to stimulate neurite outgrowth.

Lam et al. developed a set of bis-cysteine containing peptide-cross-linked HA-based hydrogels with various degrees of stiffness modified with RGD, YIGSR, IKVAV and RDG adhesive peptides [39]. They revealed that the 337 Pa (storage modulus) hydrogel was the most optimal for hiPS-NPCs (human induced pluripotent stem cell-derived neural progenitor cells) in terms of cell spreading and attachment. The authors confirmed the idea that mechanically the softest hydrogel was similar to that of native brain tissue. These results were supported by the study of Tarus et al., who used PEG-bis(thiol)-cross-linked HA hydrogels with and without RGD peptide to study their effects on mouse hippocampal neural progenitor cells (HNPCs) [40]. These authors found that neurite density was highest in the softest gel with a storage modulus of 400 Pa, and that neurites extended deeply into this soft HA-based hydrogel even in the absence of RGD.

Zhang et al. developed a two-layered system of HAMA hydrogel to mimic natural brain development [41]. The top layer consisted of hydrogel impregnated with astrocytes and the bottom layer contained hiPS-NPCs encapsulated into HAMA. The system demonstrated significant NPC migration towards the astrocyte layer. The system induced neuronal differentiation, resulting in the appearance of mature neurons within 3 weeks. Another HAMA hydrogel study carried out by Wu et al. demonstrated that stiff hydrogel variant with compressive modulus of 1.41 kPa restricted spontaneous neural differentiation of hiPS-NPC spheroids, while the soft variant (510 Pa) promoted intense neural differentiation and neurite outgrowth [42].

#### 2.1.2 In vivo studies

In 2007 Ma et al. cell-free EDC-cross-linked HA hydrogels modified with Nogo receptor antibody (NgR-Ab) were implanted into middle cerebral artery occluded (MCAO) rat model [43]. Authors found that non-modified HA hydrogel did promote regeneration, but clearly less efficiently than NgR-Ab-modified group; these results were also confirmed by the behavioral tests.

Wei et al. studied *in vivo* compatibility of IKVAV-modified EDC-cross-linked HA hydrogel [44]: an experimental rat traumatic brain injury group received the hydrogel implantation into unilateral cortex lesion site, while the control group was left untreated. HA was again proved to be highly biocompatible as there were no cavities or scarring in the treatment group 6 weeks after implantation. HA formed a highly porous network and merged with the host tissue.

In 2009, Lin et al. carried out a similar experiment to Ma et al., but without the NgR-Ab modification [45]. The control group was treated with normal saline. This study revealed HA as an effective glial scar formation inhibitor reducing astrocyte proliferation and migration.

Ju et al. investigated the effects of EDC-cross-linked HA hydrogel impregnated with mouse embryonic NSCs alone or of a HA-PLGA composite impregnated with cells and loaded with VEGF and Ang1 (angiopoietin-1) in MCAO murine model [46]. Both hydrogels were also covalently modified with NgR-Ab. Animals received intracortical implantation of the hydrogels. The results showed that both HA hydrogels fused with host tissue and remained stable for 10 weeks after MCAO. Anti-gliosis and anti-inflammatory effects were observed with functional improvement for both animal groups.

In 2014, Lam et al. studied *in vivo* structural features of bis-cysteine containing peptide-cross-linked HA hydrogel covalently modified with RGD [14]. One week after photothrombotic cortical stroke, mice were injected with 100,000 hiPS-NPCs with or without the HA-based hydrogel. One week later, immunohistochemical analysis indicated that the softer HA-based hydrogels with a storage modulus of 300 Pa induced a lower inflammatory response. The authors suggested a shielding role of HA against the host immune system. Such a HA-based hydrogel promoted neuronal differentiation of the cells but did not improve cell viability post-transplantation.

In 2016, Moshayedi et al. performed another *in vivo* study for the four distinct bis-cysteine containing peptide-cross-linked HA-based hydrogels: “HA” hydrogel (plain HA with an insensitive to MMPs [matrix metalloproteinases] cross-linker), “HA Nopt” (non-optimized), “HA Min”, and “HA Max” with varying concentrations of bioactive peptides (IKVAV, YIGSR, and RGD) [47]. These hydrogels were impregnated with hiPS-NPCs and injected into the mouse brain one week after a modeled photothrombotic stroke. Immunohistochemical staining two to six weeks after the stroke induction showed no significant differences between all groups in terms of glial response, as evidenced by GFAP and Iba1 immunostaining, and iPS-NSCs differentiation. This data suggests that HA is capable of stimulating neuronal differentiation of hiPS-NPCs regardless of additives.

Collectively, the overwhelming evidence proves that HA-based hydrogels holds great promise for brain injury repair: HA is injectable, biocompatible, biodegradable, porous, ubiquitously present in the CNS and it provides the foundation for building sophisticated hydrogel systems. It does not, however, contain any cues that would account for the interaction with transplanted cells and host tissue. The ability to direct differentiation is still arguable and can be predetermined by mechanical properties of the hydrogel. Overall comments on HA-based hydrogels in the brain tissue regeneration are summarized in Supplementary table S1.

### 2.2 Collagen type I

Collagens are extracellular matrix (ECM) proteins self-assembling into triple polypeptide helices [8]. Collagen type IV is widely presented in the adult nervous system where it forms basement membranes of the blood-brain barrier (BBB) and neuromuscular junctions [48]. Collagen IV is also a component of Matrigel (see Subsection 2.6.). Collagen type I supports axonal growth and guidance in neural development, and in adults resides in the basal lamina of the subventricular zone [49]. Collagen type I also forms *dura mater* and leptomeninges and is routinely obtained from rat tails, porcine and bovine skin [50]. Due to its role in CNS development this protein is considered to be a good candidate for brain tissue regeneration [8,15,17,20,21,29,48,[50], [51], [52]].

#### 2.2.1 In vitro studies

In 2007, Brannvall and co-workers developed a two-component collagen type I-HA (1:1 volume mix) matrix to enhance the differentiation of mouse embryonic, postnatal, and adult NS/PCs (heterogeneous cell population of neural stem and progenitor cells) [49]. The collagen network provided stability for HA and altogether presented favorable conditions for neuronal differentiation in postnatal cell culture: the cells exhibited terminal differentiation with different signaling properties and established synaptic contacts within the scaffold.

Bozkurt et al. compared a collagen scaffold with a fibrin hydrogel based system for the ability to support neurite outgrowth in the rat dorsal root ganglion (DRG) [53]. Although DRG studies deal with the peripheral nervous system (PNS), they can provide valuable data for regeneration of the relevant adult neurons. Thus, the study on DRG by Blewitt et al. [54] revealed that hydrogels with low collagen concentrations (0.4–1.0 mg/mL) helped to achieve the longest neurite extension. Hydrogels with higher collagen concentration and thus higher stability required modifications with adhesive recognition peptides to exhibit similar properties.

Deister et al. studied the effects of the collagen-HA gel on rat DRG neurite extension [55]. In contrast with study by Bozkurt et al., this research showed that neither collagen, nor HA concentration had effects on neurite outgrowth and length, thus requiring modifications.

In 2009, Kofron et al. proceeded to guide neuritis using the surface patterning of hydrogels with laminin- and chondroitin sulfate proteoglycans [56]. By placing the DRG layer between collagen type I gel and either laminin- or proteoglycan-covered glass, authors confirmed laminins micropatterning ability to guide DRG neurites on the surface of collagen. In contrast, proteoglycan patterning did not lead to extension of DRG neurites.

In 2009, Hiraoka et al. investigated collagen-based hydrogel effects on rat fetal NSCs and found that ~80% of NSCs died within two days by the anoikis mechanism [57]. Modification of collagen with laminin-derived peptides increased cell survival and stimulated maturation. In contrast, in 2010 Yao et al. reported the ability of collagen-based hydrogel to support neurite outgrowth in rat pheochromocytoma cells (PC12) without modifications; however, the laminin gradient was responsible for directing neurite growth [58].

In 2011, Lee et al. investigated the abilities of laminin- and fibronectin-modified collagen to stimulate neuro-induction of rat BMSCs (bone marrow-derived mesenchymal stem cells) [59]. The resultant gel induced neural development of BMSCs without the use of chemical differentiation factors, presumably due to the low stiffness of the three-dimensional collagen microenvironment mimicking native brain tissue.

In 2012, Swindle-Reilly studied dissociated chick DRG cells in the collagen gel modified with laminin [60]. The group revealed that low-concentrated unmodified collagen hydrogels (0.4 – 1.5 mg/mL) supported neurite growth more so than the modified variant.

In 2013, Koutsopoulos et al. compared viability and differentiation of the mouse adult NSCs in peptide nanofiber hydrogels, Matrigel, and collagen and reported the inferior properties of collagen in contrast to the studied gels [61].

#### 2.2.2 In vivo studies

In 2010, Yu et al. implanted MCAO rats with commercially available 3D porous collagen sponges containing rat embryonic NSCs [62]. Although present but not in the form of a hydrogel, collagen was shown to be neuro-compatible.

Zhong et al. carried out a study in a photothrombotic stroke murine model [63]. The mice were implanted with mouse embryonic NPCs encapsulated in HA-heparin-collagen hydrogel. The scaffold did not swell, protected graft cells from microglia and macrophages and increased survival of NPCs in the hostile environment. No effect on cellular differentiation and migration was observed when compared to control cells with no hydrogel.

Hoban et al. provided evidence for collagen type I as a non-cytotoxic and self-healing hydrogel *in situ*. The group injected the striatum of sham rats with glial cell line-derived neurotrophic factor (GDNF)-overexpressing rat bone marrow MSCs encapsulated in a collagen hydrogel cross-linked with 4S-StarPEG (PEG ether tetrasuccinimidyl glutarate) [64]. The hydrogel prevented micro- and macrogliosis compared to the medium-injected control group. However, the scaffold poorly supported MSCs survival and decreased in volume several days post gelation *in vitro* and *in vivo*.

Liang et al. developed a set of hydrogels with variable HA:gelatin:PEG diacrylate ratios and used them to encapsulate C17.2 NSCs (mouse immortalized NSCs), ReNcells (human immortalized NPCs), and GRPs (human glial-restricted progenitor cells) [65]. This group found that an increase in gelatin content extended the gelation time and that HA promoted survival of all cell lines. In contrast, gelatin promoted survival and proliferation only in ReNcells and C17.2 cells. The hydrogels suppressed the innate immune response and improved viability of ReNcells injected into the striatum of immunodeficient mice.

Nakaji-Hirabayashi and co-workers studied the collagen hydrogel with or without laminin-derived peptides with rats *in vivo*: the group injected rat fetal NSCs encapsulated by the striatum and confirmed that collagen acted as a barrier for microglia and that modifications were required to reduce apoptosis and to promote proliferation of NSCs [66].

In conclusion, it should be kept in mind that collagen type I/gelatin-based hydrogels are not entirely physiological as collagen proteins are poorly represented in brain parenchyma. Collagen type I/gelatin-based hydrogels are injectable, biodegradable, interactive, non-swelling and can be modified with laminin to promote cell attraction. Prior to their use in clinical applications, collagen and gelatin-based hydrogels should be thoroughly tested for immuno-/allergenicity due to their xenogenic nature. Overall comments on collagen type I hydrogels in brain tissue regeneration are summarized in Supplementary table S2.

### 2.3 Alginate

Alginate is a natural polysaccharide composed of β-D-mannuronic acid (the “M” residues) and α-L-guluronic acid (the “G” residues), produced by brown algae and some bacteria [67,68]. The hydrogel is typically produced via inter- and intrachain cation (typically by Ca^2+^) cross-linking [69]. Alginate hydrogels are non-biodegradable in mammalian brain as long as they do not produce alginases [68]. For recent reviews see [9,13,15,19,67,70].

#### 2.3.1 In vitro studies

In 2007, Ashton et al. looked if they could increase the rate of degradation for the alginate hydrogel without harming rat NPCs [68]. To do this, they incorporated alginate lyase-loaded PLGA microspheres into the hydrogel. The study revealed that the NPC proliferation was positively influenced by the rate of alginate degradation. Non-degradable control hydrogel negatively influenced cell morphology and decreased the ability of NPCs to penetrate through the cross-linked hydrogel. Degraded alginate products did not induce cell death but at high concentrations could restrict the proliferation of NPCs.

In 2009, Purcell et al. studied a set of alginate hydrogel beads with high “G” residues, high “M” residues, “G/PLL” hydrogel beads (PLL-coated “G” beads), and “M/PLL” beads (PLL-coated “M” beads) [71]. Each hydrogel bead type was also loaded with NGF (nerve growth factor), GDNF (glial cell derived neurotrophic factor), or BDNF (brain-derived neurotrophic factor) and evaluated with mouse embryonic NSCs. While NSCs survived and proliferated on either hydrogel type, high “G” hydrogel beads were more mechanically stable than the “M” type, thus providing potentially longer isolation from immunity of the host.

Banerjee et al. investigated the influence of alginate hydrogels with stiffness (elastic modulus) of 0.18, 1.03, 1.73, and 19.7 kPa on proliferation and differentiation of rat adult NSCs [72]. The softest hydrogel stimulated proliferation and differentiation of cells greater than the other types. Eftekharzadeh et al. assessed alginate antioxidant properties [73] using NT2 cell line (human NPCs) and claimed that alginate had the ability to protect the cells against H_2_O_2_, preventing apoptosis via inhibiting caspase-3 activation and increasing synthesis of the heat shock proteins Hsp-70, HO-1 protein (Heme oxygenase-1), γ-GCS (γ-glutamyl cysteine synthetase), and Nrf2 (Nuclear factor [erythroid-derived 2]-like-2 factor). Alginate also inhibited amyloid β formation, thus demonstrating a cytoprotective role *in vitro*.

In 2011, Frampton et al. developed a safe and fast method for fabrication of alginate constructs [74] and applied it to an alginate-cell suspension with embryonic rat hippocampal neurons, LRM55 rat astroglioma cells, rat cortical astrocytes, and rat microglia cells. Alginate was functionalized with a mixture of RGD, IKVAV peptides, and laminin before the experiment. This micromolding fabrication method minimized exposure of the cells to potentially toxic Ca^2+^ ions, resulting in accelerated maturation of neural cells and increased synaptic electrical activity of neurons. However, cell migration in these scaffolds was restricted, possibly due to insufficient alginate porosity, in agreement with results of Ashton et al. [68].

Matyash et al. investigated behavior of rat and human NSCs on soft alginate hydrogels cross-linked with sub-stoichiometric concentrations of Ca^2+^, Ba^2+^, and Sr^2+^ ions, with respect to the varying “G” and “M” content [75]. Authors provided evidence for the ability of non-functionalized alginate to support neural adhesion and neurite outgrowth. The group also demonstrated that alginate had antioxidant neuroprotective properties and that neurons preferred a soft matrix. Moreover, low alginate viscosity was found to be non-detrimental to neurite outgrowth while high “G” alginate hydrogel was deemed less permissive to neurites.

Kuo et al. reported the alginate-γ-PGA (poly[γ-glutamic acid]) hydrogel-based inverted colloidal crystal (ICC) scaffold [76]. The group also modified the scaffold with a TATVHL peptide to induce cell adhesion. The scaffold was highly porous, non-cytotoxic, and promoted neuronal differentiation of mouse iPS-NPCs better than the control. The morphology of the scaffold depended on Ca^2+^ concentration and the degree of cross-linking, while the TATVHL peptide accelerated neuronal differentiation of iPS-NPCs.

In 2015, Tseng et al. investigated the physical properties of alginate hydrogels and its interactions with mouse NSC spheroids [77]. The group reported a number of limitations , making alginate unsuitable for use in mouse NSC cultures: the hydrogel was non-self-healing, it was difficult to inject, fragmenting after each injection, and slow to recover after strain-induced stress. Most of these results contradict with the results by Bozza et al. [78] (see below).

#### 2.3.2 In vivo studies

Ciofani et al. demonstrated alginate as a suitable matrix for *in vivo* delivery systems [69]. Tests were performed in mice, which were implanted with Ca^2+^-cross-linked alginate doped with WFA (*Wisteria floribunda* agglutinin), labeling perineuronal areas in rat brain [79]). Alginate served as a suitable delivery system forming an interface between the implant and host tissue.

In another delivery study published by Emerich et al., either VEGF-containing or blank alginate hydrogel was injected into the rat striatum, followed by treatment with quinolinic acid to mimic the onset of Huntington’s disease [80]. Alginate provided sustained delivery of VEGF, thus preventing development of the disease however no morphological or functional recovery was observed in the control groups, similarly to results produced with MCAO rat model [81]. Collectively, alginate-VEGF system proved to be suitable for neuroprotection and neuroregeneration.

Bozza et al. studied survival and differentiation of mouse ESCs (embryonic stem cells) and embryonic NSCs in blank alginate, or modified with laminin, RGD peptide and HA beads [78]. The survival of ESCs was satisfactory in every type of hydrogel, however 1% alginate hydrogels supported the highest cell viability and rates of differentiation. Moreover, ESCs cultured in alginate-HA beads formed rosettes and showed robust neurite growth. Similar results were acquired using murine NSCs, where alginate was injected into the striatum of MCAO mice. Alginate was biocompatible and did not induce inflammation in mice, although functional tests were not carried out. In addition, alginate showed sufficient *in situ* polymerization contrary to the study by Tseng et al. [77].

In conclusion, the disadvantages of alginate-based hydrogels seem to outweigh the advantages, however they do not fulfill the basic requirements for brain tissue regeneration: self-healing, porosity, and biodegradability. This limits the promise of alginates for brain tissue repair. However, existing *in vivo* data still attracts research interest to this material, especially in terms of further modification with HA or other moieties. Overall comments on alginate hydrogels in brain tissue regeneration are provided in Supplementary table S3.

### 2.4 Chitosan

Chitosan is a natural polymer produced by the deacetylation of crustacean chitin N-acetyl-glucosamine residues [15,82]. It is built of randomly bound β-(1-4) D-glucosamine links and N-acetyl-d-glucosamine. For recent reviews see [9,10,12,[15], [16], [17],[19], [20], [21],^83^].

#### 2.4.1 In vitro studies

In 2007, Crompton et al. studied a PDL (poly-D-lysine) modification of chitosan/glycerophosphate salt hydrogel and its interactions with mouse fetal cortical cells [84]. The hydrogel was found to be suitable for these cells: the modification with PDL promoted survival of the cells and neurite outgrowth. However, the chitosan hydrogel needed a long gelling time (30 min), which led to cell sedimentation.

Yu et al. modified with adhesive peptides the thiolated ammonium persulfate/sodium metabisulfite-cross-linked methacrylamide chitosan (MAC) and studied its effects on rat postnatal superior cervical ganglion neurons [85]: they found that the high porosity of the chitosan hydrogel was negatively affected by the cross-linking; the intrinsic chitosan cell-adhesiveness was insufficient and required improvement; lastly, even methacrylated chitosan was biodegradable by the lysozyme.

In 2009, Cao et al. investigated bioactivity in chitosan-agarose co-gel [82] and revealed that at pH > 6.5 chitosan chains aggregated when combined with agarose. However, either co-gel formulation was better than unmodified agarose hydrogel for adhesion of chick embryonic cortical neurons. Chitosan also slightly decreased the stiffness in the co-gel system and chitosan:agarose ratio positively correlated with branching of neurites.

In 2009, Leipzig et al. investigated the effects of photocross-linked (2,2-dimethoxy-2-phenylacetophenone photoinitiator) MAC stiffness on behavior of rat adult NS/PCs [86]. The group defined the Young’s modulus of 3.5 kPa to be optimal for NS/PCs proliferation. The softest hydrogel of < 1 kPa supported all three neural tissue lines (neurons, astrocytes, and oligodendrocytes), while the stiffest hydrogel of 7.0 kPa supported oligodendrocyte differentiation. However, the observed effects could only be true for rat NS/PCs. In 2010 authors reported the dose-dependent rat NS/PC maturation [87] in response to the MAC modification with IFN-γ. In another work, the authors used for modifications the RGD peptide [88].

In 2011, Zhang et al. modified chitosan via PEGcross-linking [89]. The modification improved self-healing and resistance to degradation with lysozyme, however the hydrogel could be hydrolyzed by the protease papain. In 2015, a similar study was carried out by Tseng et al. [77].

Kuo et al. developed a chitosan-gelatin hydrogel-based ICC scaffold for rat BMSCs [90]. The group also modified the scaffold with laminin-derived peptides to induce neuronal differentiation of BMSCs. Obtained chitosan-based scaffolds were as a result highly porous. BMSCs adhered to the scaffold, yet chitosan-gelatin hydrogel supported insufficient neuronal differentiation without modification with peptides. An increase in chitosan content increased cationic cytotoxicity.

In 2016, Wei et al. developed a CEC-l-OSA hydrogel (N-carboxyethyl chitosan cross-linked by oxidized sodium alginate) and investigated its physical behavior and interactions with rat fetal NSCs [91]. Briefly, the hydrogel was self-healing under physiological conditions, stable, and exhibited cytocompatibility. NSCs proliferated slower in 3D culture when compared to 2D, but exhibited neuronal differentiation in 3D. On the other hand, in 2D culture, cells tended to differentiate into astroglial phenotype.

#### 2.4.2 In vivo studies

Tseng et al., exposed zebrafish embryos to ethanol and subsequently injected them either with PBS (phosphate-buffered saline), chitosan-based hydrogel alone, chitosan gel with dispersed NSCs, chitosan with neurospheres, or cell suspension with no gel [77]. The neuroprotective effects were assessed through specific movements, spontaneous contractions, and hatching of the embryos. Animals that received chitosan-neurosphere system injection showed improved functional recovery.

In conclusion, chitosan- and chitosan/agarose-based hydrogels show promising physical properties in terms of shear-thinning and self-healing but seem to possess poor neuro-interactive cues, i.e. they poorly stimulate the neurite outgrowth. However, this can be improved by modifying the mechanical properties of hydrogel and by coupling it with IFN-γ or RGD (poly)peptides. Chitosan biodegradability definitely requires further investigation. Overall comments on chitosan- and agarose-based hydrogels in brain tissue regeneration are summarized in Supplementary table S4.

### 2.5 Methylcellulose

Methylcellulose is a compound derived from cellulose polysaccharide via substitution of hydroxyl groups with methoxide groups [15,17]. While cellulose is a solid material, methylcellulose exhibits low viscosity as a fluid at room temperature and gels at 37 °C [15]. In most recent studies, methylcellulose was used in a co-gel with HA (HAMC). This is reflected in this review. For recent reviews see [10,15,[17], [18], [19]].

#### 2.5.1 In vitro studies

In 2009, Wang et al. investigated HAMC hydrogel as a drug delivery system [92]. The group provided evidence that methylcellulose can increase solubility of hydrophobic and other poorly soluble drugs within the hydrogel. The study suggested that HAMC hydrogels could be tuned in order to deliver various drugs and to modulate drug release profiles.

Another drug-delivery approach was proposed by Baumann et al. [93]. They developed a series of HAMC blends loaded with PLGA particles. The hydrogel was injectable and self-healing but was swelling over time. This could be ameliorated at higher concentration of methylcellulose and use of lower concentration of HA.

In 2010, Hsieh et al. produced a composite of HAMC hydrogel and electrospun fibers based on of either collagen or poly(ε-caprolactone-co-d,l-lactide) [94]. The group investigated its effects on rat adult NS/PCs *in vitro* and in ‘simulated *in vivo’* conditions. HAMC hydrogel with cells was injected through a needle into the 96-well culture plates. The hydrogel prevented cell sedimentation and aggregation and supported NS/PCs viability, but it did not promote cell differentiation unless present in a composite with poly(ε-caprolactone-co-D,L-lactide) fibers.

In 2012, Stanwick et al. performed a drug delivery study on HAMC with PLGA nanoparticles loaded with NT-3 (neurotrophin-3) [95]. Tam et al. investigated HAMC as a vehicle to deliver adhesive peptides and rPDGF-A (recombinant platelet derived growth factor subunit A) bound to methylcellulose via maleimide-streptavidin-biotin chemistry [96]. The studies provided substantial evidence that HAMC can be an important part of injectable drug delivery systems.

#### 2.5.2 In vivo studies

In 2010, Baumann et al. performed intrathecal delivery of HAMC hydrogel with incorporated non-drug-containing blank PLGA nanoparticles in rats [97]. This work provided further evidence on HAMC being a perspective clinical CNS drug delivery platform. Thus, it was found that nanoparticles increased the hydrogel stiffness. Both blank and modified with nanoparticles HAMC acted like aCSF (artificial cerebrospinal fluid) in terms of macro- and micro-gliosis.

In a brain regeneration study by Wang et al., HAMC-borne erythropoietin was delivered epicortically in stroke-induced mice [98]. The study pointed at insufficiency of unmodified HAMC hydrogel in stimulating neuroregeneration as it induced host cell proliferation and reduced host cell apoptosis only in the erythropoietin-modified form. Untreated animals showed no significant differences from animals treated with blank HAMC. HAMC alone reduced the presence of microglial marker CD68 and astroglial marker GFAP. The same group of authors also utilized EGF- (epidermal growth factor) or PEG-EGF for modification of HAMC [99]. In 2013, this team published another study on sequential epicortical delivery of EGF-PEG and erythropoietin in this model [100]. EGF-PEG was encapsulated into PLGA nanoparticles and erythropoietin was encapsulated into poly(sebaric acid)-coated PLGA nanoparticles. Such sequential delivery demonstrated promising results for anti-apoptotic protection, proliferation, and differentiation of host NS/PCs. Blank HAMC again showed anti-inflammatory and anti-gliosis effects.

In 2012 Austin et al. subjected rats to spinal cord compression and subsequently injected the compression sites with either HAMC or aCSF [101]. The study revealed a trend towards axonal preservation and functional improvement that could be due to lower production of cytokines and chemokines. The authors attributed the result to the HA contribution known to play a role in inflammation and tissue repair by interaction with inflammatory response-associated cells and ECM proteins.

In another brain injury repair study, Ballios et al. implanted stroke-injured mice with mouse adult NSCs encapsulated in HAMC [102]. The cells were evenly distributed throughout the implant and showed no sedimentation. HAMC system promoted NSCs survival, penetration into the host brain and functional recovery.

Pakulska et al. investigated X-methylcellulose, both thermally and chemically cross-linked, and its effects upon rat intrathecal injection [103]. The hydrogel was either blank or modified with chondrotinase ABC or PLGA nanoparticles loaded with SDF1α (stromal cell derived factor). Neither formulation of the hydrogel caused neuroinflammation and astrocytosis and after 8 weeks *in vivo* there were no remaining hydrogel present *in situ*.

In conclusion, methylcellulose has been extensively studied in two-component HAMC hydrogel systems that benefit from the pure HA hydrogels. The methylcellulose-based system does not possess any significant drawbacks and is promising for brain regeneration: methylcellulose-based hydrogels are injectable, biocompatible, biodegradable, porous, and non-selling. The only drawback is a limit of its further modification. Overall comments on methylcellulose and HAMC as candidates for brain tissue regeneration are presented in Supplementary tables S1 and S5.

### 2.6 Matrigel

Matrigel is a mixture of extracellular factors extracted from sarcomas of Englebreth-Holm-Swarm mice, also referred to as EHS gel. It is rich in growth factors and extracellular proteins including collagen type IV and laminin [15,20,104]. This mixture is highly advantageous for *in vitro* studies but use in humans is unlikely due to its non-standardized composition and possible carcinogenicity. For recent reviews see [15,20,22,26,105].

#### 2.6.1 In vitro studies

In 2007, Ju et al. investigated the effects of salmon fibrin gel on mouse embryonic spinal and cortical and rat embryonic cortical neurons. Matrigel cultures served as a control [106]. The study revealed inferior activity of Matrigel and human and bovine fibrin samples for neurite growth when compared to salmon fibrin.

In 2008, Ma et al. published a study on human embryonic stem cell (hESC) neural differentiation on 2D surfaces coated with PDL, PDL/laminin, PDL/fibronectin, collagen type I, or Matrigel [107]. Neural differentiation and neurite growth were more intensive on laminin-rich substrates including Matrigel.

Thonhoff et al. investigated viability and differentiation of human fetal NSCs (hNSCs) on Matrigel and ‘PuraMatrix’ – a 16-mer consisting of four RADA peptides, also known as RADA16-I [108]. Matrigel stimulated astroglial differentiation of hNSCs, possibly due to its toxic effects on neurons. Surprisingly, Matrigel decreased cell viability at a concentration of 10%, but had no effect at 50%. The authors speculated that astroglial expansion was induced by neuronal death at the initial phase followed by the proliferation of astrocytes stimulated by growth factors in the increasing concentration of Matrigel. RADA16-I was clearly superior to Matrigel.

In 2010, Uemura et al. investigated effects of growth factor-reduced Matrigel on the survival and differentiation of mouse ES-NPCs (embryonic stem cell-derived neural progenitor cells) [109]. Other matrices tested included collagen type IV, ornithine/laminin, and RADA16-I. In that study, Matrigel supported the viability, migration, and maturation of NPCs much more efficiently.

In 2013, Koutsopoulos et al. investigated the viability and differentiation of mouse adult NSCs in peptide nanofiber hydrogels, Matrigel, and collagen [61]. Matrigel supported poor survival of NSCs, but still performed better than collagen type I hydrogel and in inducing neuronal differentiation. However, two weeks later, the peptide nanofiber hydrogels showed better cell survival.

In studies of PNS, Dewitt et al. reported the Collagen I-Matrigel composite scaffold for maturation of rat primary Schwann cells [110]. Matrigel increased the elastic modulus of the scaffold by more than 1.5-fold A 2016 study by Sun et al. investigated the effects of Matrigel on mouse postnatal spiral ganglion neurons [111] and found positive effect of Matrigel on cell viability and the maturation of neurons.

#### 2.6.2 In vivo studies

The Uemura group [109] used a murine model for intastriatal injection of ES-NPCs (embryonic stem cell-derived neural progenitor cells)-rich Matrigel into one hemisphere and ES-NPCs-rich differentiation medium into contralateral striatum. The results showed that Matrigel had an anti-inflammatory effect, promoted NPCs maturation in dopaminergic neurons and host cell proliferation.

In 2010, Jin et al. injected MCAO rats with human NS/PCs encapsulated in and cultured within Matrigel [112], which stimulated maturation of the cells and promoted structural and functional recovery. However when cells were implanted without Matrigel, or Matrigel was injected without cells, authors observed negative results.

Before it can be translated into a clinical setting Matrigel has to be proven not to be carcinogenic. Overall comments on Matrigel as candidate for brain tissue regeneration are presented in Supplementary table S6.

### 2.7 Fibrin

Fibrin is a natural enzymatically degradable protein involved in blood and lymph clotting following injury [15], produced by partial lysis of fibrinogen by thrombin [21]. As long as it is obtained from the autologous blood donor, it is highly biocompatible [15,113]. However, either fibrin itself or increased plasmin activity can induce neuroinflammation [114,115]. For the past decade it has been extensively studied in terms of SCI and PNS repair [[15], [16], [17],^21^].

#### 2.7.1 In vitro studies

In a fibrin-assisted PNS regeneration study [116], fibrin was found to be a suitable NGF delivery system, demonstrating the release in dependence of the gel stiffness. In another study authors demonstrated the advantage of fibrin modification with NGF-binding peptide in order to enhance the neurite extension in chick embryonic DRG cells [117].

In an SCI study by Willerth et al., murine ES-NPCs were cultured in fibrin scaffolds in the presence of several growth factors: Shh (sonic hedgehog), NT-3, bFGF (basic fibroblast growth factor), PDGF (platelet-derived growth factor), and CNTF (ciliary neurotrophic factor) [118]. The ‘cocktails’ containing minimal amounts of growth factors promoted neuronal and oligodendrocytal differentiation in cells and in their absence differentiated predominantly towards an astroglial phenotype.

Sarig-Nadir et al. investigated effects of fibrin and collagen composition on chick embryonic DRG cells [119]: modification of fibrinogen reduced neurite length proportionally to PEG content. The neurites exhibited an abnormal morphology in PEGylated collagen: they did not branch, compared to the fibrin gels.

Mooney et al. clearly show that fibrin did not induce apoptosis and promoted neuronal differentiation and maturation of rat fetal NSCs [120]: all fibrin matrices with varying stiffness supported an increase in cholinergic and dopaminergic neurons and inhibited glial differentiation. Interestingly, increases in stiffness promoted growth of dopaminergic neurons and glial cells. The stiffness was controlled by varying the ratios between the fibrinogen and/or thrombin.

In 2011, Man et al. studied mouse neonatal DRG behavior in fibrin [121]. Increases in NaCl and/or fibrinogen concentrations resulted in formation of a stiffer matrix with smaller pores and inhibited neurite outgrowth. The gel was shown to be susceptible to cellular MMPs.

#### 2.7.2 In vivo studies

In 2009, Itosaka et al. injected hemisected rat spinal cords with mouse BMSCs encapsulated in fibrin matrix [113]. Authors observed that fibrin supported survival, migration, and neuronal differentiation of BMSCs. In another SCI study by Hyatt et al., fibrin was demonstrated to be a suitable enzyme delivery system [122]: lesioned rat spinal cord received an injection of chondrotinase ABC-loaded fibrin hydrogel. Fibrin provided prolonged activity of chondroitinase ABC and effectively prevented GAG (glycosaminoglycan) infiltration into the site.

In conclusion, fibrins are injectable, biocompatible, biodegradable, porous, and non-swelling hydrogels. Fibrin shows one clear advantage over other types of protein hydrogels for nervous system repair: it can have complete biocompatibility when used autologously. Precise control of its mechanical properties enables neuronal differentiation. These characteristics make fibrin an interesting subject for further tissue engineering studies but so far fibrin hydrogel systems have been poorly implemented in brain studies (See supplementary table S7).

### 2.8 Gellan Gum

Gellan gum is a natural linear polysaccharide produced by *Pseudomonas elodea*, based on monomer unit of D-glucose, L-rhamnose, and D-glucuronic acid [16,123]. In food industry it is also known as a stabilizer and thickening agent E418 [124]. For recent reviews see [16,123,125].

#### 2.8.1 In vitro studies

In 2007, Smith et al. proposed gellan gum as a tissue engineering material [124] and found that gellan gum gelled by divalent cations was injectable. In 2012, Silva et al. investigated Gellan Gum effects on rat adult NS/PCs [126]. The study revealed the benefit from modification of gellan gum with RGD peptide in order to provide interface sites for cells and enhance neurite extension. In both modified and unmodified gels, most NS/PCs differentiated into oligodendrocytes. RGD modification prevented cells from forming aggregates prone to apoptosis. The co-culturing NS/PCs with olfactory glial cells increased proliferation but not differentiation.

In 2015, Lozano et al. presented a 3D mammalian brain cortex tissue model printed with RGD-modified gellan gum [127]. The model consisted of rat primary cortical neurons arranged in six layers. The gel supported cell viability, differentiation, and promoted neurite invasion into neighboring layers. Thus, RGD-modified gellan gum protected the cells during the process of hand-held bioprinting. However, neurons did not respond well to the unmodified gel. In addition, gellan gum needed purification from divalent cations to perform injections during bioprinting. In 2015, Ferris et al. assessed PC12 cell line survival, growth, and normal function in the RGD-modified gellan gum [128]. The latter cues were all retarded in the unmodified gellan gum.

In 2017, a study on gellan gum cross-linked with poliamines and its compatibility with hiPS-NSCs was published by Koivisto et al. [129]. Spermine and spermidine were used as cross-linkers, and higher SPM or SPD content resulted in the increase of compressive moduli of the gels from approximately 3.5 kPa (0.40% Spermine) to approximately 22.5 kPa (3.00% Spermidine). Spermine-cross-linked gellan gum demonstrated typical hydrogel behavior (G’>G”). Spermidine caused very rapid gelation, the result being the formation of nucleating cross-linking spots, and at the same time supported highest rate of neurite outgrowth. All hydrogel types were shown to be non-toxic to hiPS-NSCs.

Collectively, gellan gum is a novel biomaterial in neuroregeneration studies but so far it has been poorly implemented *in vitro* and was not yet studied in *in vivo* (See Supplementary table S8).

### 2.9 Self-assemblingpeptide/protein-based hydrogels

The hydrogels made of self-assembling peptides are synthesized from naturally occurring and reusable amino acids. They are commonly arranged into di- [130], tri- [131], and tetra-block [104] amphiphilic copolymers. They self-assemble in water into 3D structures with hydrophobic core and hydrophilic outer surface when exposed to millimolar concentrations of monovalent cations [15,104]. Self-assembling peptides are reviewed in this subsection along with some protein-based hydrogels. For recent reviews see [10,15,17,21,[132], [133], [134], [135]].

#### 2.9.1 In vitro studies

In 2007 Fisher et al. genetically engineered two triblock protein hydrogels: unmodified CRC protein (“R” is for random coil middle block and “Cs” are for ampholytic leucine zipper flanking domains) and CRC-RGDS. RGDS adhesive peptide was inserted into the R domain [131]. They tested the peptides physical behavior and the impact on rat adult hippocampal NSCs. Both proteins had identical secondary structure. The proteins were more stable in water when compared to the culture medium. *In vitro* studies showed greater adhesion, distribution, and proliferation of rat NSCs cultured on RGDS-modified substrate.

In 2008, Nakaji-Hirabayashi et al. a genetically engineered chimeric protein consisting of a long R-helical polypeptide, keratin-14 (K14) and globular domain 3 of laminin R3 chain (LG3) incorporated into α-keratin hydrogels [136]. The hydrogel self-assembled via coiled-coil domains. Pure keratin, keratin/LG3K14, and keratin/K14 hydrogels similarly supported viability of rat fetal NS/PCs. The modification with LG3K14 was shown to significantly improve cell adhesiveness, differentiation, proliferation, and distribution. Few drawbacks of this system included the need for dissolving keratins by strong denaturants such as urea or thiourea and the lack of knowledge on their *in vivo* degradation.

In 2009, Koutsopoulos et al. studied drug-releasing properties of the RADA16-I hydrogel loaded with various functional proteins including lysozyme, soybean trypsin inhibitor, BSA (bovine serum albumin), and IgG [137]. The results showed that the peptide did not alter secondary and tertiary structures and the proteins remained functional; the release kinetics was tunable by changing the peptide nanofiber density.

In 2010, Gelain et al. investigated the release of bFGF, VEGF, and BDNF from the delivery systems based on RADA16-I peptide, RADA-DGE (RADA16-I modified with GGDGEA peptide motif), or RADA-PFS (RADA16-I modified with GGPFSSTKT peptide motif) [138]. The release characteristics depended on the charge correlations. RADA16-I was shown to be less negatively charged than RADA-DGE and less positive than RADA-PFS, while VEGF was negatively charged and bFGF and BDGF were positively charged. The drastic charge differences in hydrogel-drug systems resulted in a prolonged and incomplete drug release. The functional activity of bFGF released from RADA16-I and RADA-DGE hydrogels was confirmed with the mouse adult NSCs.

Ortinau et al. investigated the effects of RADA16-I hydrogel and its modification with laminin using human NPCs [139]. The modification induced cell differentiation and maturation into dopaminergic neurons and prevented aggregation of the NPCs. RADA16-I with a laminin concentration of 0.25% induced the highest dopaminergic maturation of the neurons.

In 2010, Zhang et al. proposed a modification of RADA16-I with IKVAV [140]. The group examined three different matrices: pure RADA16-I, RADA16-IKVAV, and their combination called IKVAVmx with neonatal mouse NSCs. IKVAVmx hydrogel was found to be the most beneficial in terms of cell proliferation, distribution, migration, neuronal lineage differentiation, and maturation.

In a PNS study by Li and Chau, the IKVAV peptide was tested with three following matrices: RADA16-I termed as (RADA)_4_, (RADA)_3_IKVAV(RADA)_3_ termed as _3_IKVAV_3_, and (RADA)_4_-IKVAV termed as _4_IKVAV [141]. The modification of the original hydrogel did not show significant effect on its structure. IKVAV modification enabled more intense neurite outgrowth, neural differentiation, maturation, and improved viability of PC12 cells. The highest viability, proliferation, and maturation were observed with _3_IKVAV_3_ matrix.

In 2010, Taraballi et al. developed RADA16-I matrices modified with another functional biological motif, PFSSTKT, also known as BMHP1 – bone marrow homing peptide 1, with or without glycine spacers [142]. The resultant peptides included RADA16-I-PFSSTKT termed as 0G-BMHP1 (no glycine spacers), RADA16-I-GGPFSSTKT termed as 2G-BMHP1 (two glycine spacers), and RADA16-I-GGGGPFSSTKT termed as 4G-BMHP1 (four glycine spacers). The group studied the structure of these hydrogels and their effects on mouse NSCs. Modifications did not significantly alter structure of the original RADA16-I matrix, but made it more stable, with the exception of the 0G-BMHP1 hydrogel. The highest cell distribution, adhesion, and survival were observed with the 4G-BMHP1 hydrogel.

In 2011, Cunha et al. compared RADA16-I hydrogels modified with RGD, BMHP1, and BMHP2 [143]. The viability of mouse adult NSCs was high in all tested matrices. However, cell proliferation was higher in the BMHP2- and RGD-modified hydrogel. RADA16-RGD stimulated the most robust differentiation. These peptide scaffolds could be also tuned by altering the concentration to meet the needs for appropriate stiffness.

In 2012, Yla-Outinen et al. characterized the lifespan of human ES-NPC grown on the top layer, under or encapsulated in RADA16-I in comparison to the laminin-coated surface [144]. The study showed that regardless of location in the matrix the RADA16-I cell survival was sufficiently high. RADA16-I stimulated differentiation and maturation of the cells; and neurite outgrowth was greater with the RADA16-I hydrogel, especially in 0.25% RADA16-I, when compared to the laminin-coated surface. The matrix was also compatible with glial cells, with the best results achieved in 0.10% RADA16-I. The neuronal networks grown from ES-NPCs inside the gel showed spontaneous electrical activity. The authors observed no benefits from functionalizing the hydrogel with laminin.

In a cultivation protocol by Liedmann et al., human NPCs were cultured on either unmodified RADA16-I or on RADA16-I-laminin [145]. The cells showed improved distribution and neuronal differentiation on RADA16-I-laminin. In 2013, Koutsopoulos et al. compared the viability and differentiation of mouse adult NSCs in RADA16-I, Matrigel and collagen-based hydrogels. RADA16-I was either blank or modified with peptides including GG-SKPPGTSS, GG-PFSSTKT, or GG-RGDS (GG is for glycine-glycine spacer) [61]. All peptide hydrogels showed superior survival of the cells and the greatest survival was observed for the GG-SKPPGTSS-modified RADA16-I. Unmodified peptide hydrogel induced weaker neuronal differentiation of cells when compared to the Matrigel.

In a PNS study by Lampe et al. (2013), hydrogels composed of elastin-like proteins were tested with chick embryonic DRG cells [146]. The proteins were also modified with either adhesive RGD or non-adhesive RDG peptides. The elastic modulus of the resultant hydrogels could be tuned by varying cross-linking density by tetrakis(hydroxymethyl)phosphonium chloride. Viability of the cells was not altered by stiffness and RGD incorporation. On the other hand, neurite outgrowth was induced by RGD incorporation and was increased in the softest hydrogels with an elastic modulus of 500 Pa.

#### 2.9.2 In vivo studies

In 2008, Sierpinski et al. published a study on peripheral nerve repair by human hair-derived keratin hydrogel using tibial nerve transection model [147]. Though being a PNS study, it provided us with important cues about keratin-based hydrogels, which are highly porous and can stimulate the neurite outgrowth and vascularization.

In 2009, Yang et al. developed a library of diblock copolypeptide hydrogels including combinations of lysine and leucine (K_x_L_y_), arginine and leucine (R_x_L_y_), glutamate and leucine (E_x_L_y_). Plain poly-lysine (K_x_) was also utilized as a non-gelling control [130]. The peptide solutions were injected unilaterally into the caudate putamen nuclei of mice. Controls received an injection of sterile saline. Physical behavior of the proteins depended on the composition: K_190_L_10_ did not form a gel *in vitro* nor *in vivo*; K_170_L_30_-0.5%, K_160_L_40_-0.25%, and K_160_L_40_-0.5% did not gel *in vivo*; K_180_L_20_-3%, E_180_L_20_-2%, and R_180_L_20_-3% poorly gelled *in vitro* but consistently gelled *in vivo*. Diblock copolypeptide hydrogels caused minimal gliosis, visible inflammation *in situ,* however no evident toxicity to neurons was detected. The gels also induced ingrowth of endothelial cells and stimulated vascularization. However, limited ingrowth of nerve fibers and neuron-supportive astroglia was observed.

Guo et al. injected RADA16-I and sterile saline as a negative control into the brain injury sites in a rat TBI model [148]. The results were promising: the cavity injected with self-assembling peptides reduced significantly in size and integrated with no obvious gaps, showed decreased astrogliosis and microgliosis. However, RADA16-I did not promote neural-lineage cell migration and migration of oligodendrocytes, in addition the protection from apoptosis was lower when compared to controls.

In 2013, Cheng et al. tested the IKVAV modification of the hydrogel. The authors injected rats with either RADA16-I or RADA16-IKVAV after traumatic brain injury [149]. The animals were divided into five groups according to the injectate: (1) RADA16-IKVAV with encapsulated rat NSCs; (2) RADA16-I with encapsulated rat NSCs; (3) plain RADA16-IKVAV; (4) plain NSCs; (5) sterile saline. Both hydrogels supported survival and promoted proliferation of the encapsulated cells. Proliferation was more prominent in the IKVAV-modified hydrogel. Neuronal differentiation, maturation, adhesiveness, distribution, neurite outgrowth was also enhanced in RADA16-IKVAV. Regeneration was significantly delayed in the hydrogel-only group.

In 2016, Francis et al. performed neuronal induction of human iPS-NPCs, encapsulated the cells into RADA16-I microspheres and injected them into the mouse striata eight days after encapsulation [104]. Pre-culturing allowed the cells to synthesize the extracellular matrix proteins including collagen and laminin, promoting neurite outgrowth and enhancing survival of the cells *in vivo*. Microspheres supported viability of the cells *in vivo* and provided excellent integration with the host tissue.

Collectively, self-assembling peptide/protein-based constructs represent the most heterogeneous group of hydrogels. Their drawbacks are easily adapted by modifications. For example, modifications of the hydrogels with IKVAV, BMHP1 or RGD peptides increase cell viability neuronal differentiation and neurite outgrowth. Their mechanical properties are easy tunable by the polymer concentration adjustment or cross-linking. *In vivo* data are also promising for the translation to a clinical setting (see Supplementary table S9).

## 3 Synthetic polymers

### 3.1 Poly(ethylene glycol)

Poly(ethylene glycol) (PEG) has been the most extensively studied synthetic hydrogel. Apart from PEG hydrogels, PEG has been widely implemented in modification of other hydrogels [7,15,150,151].

#### 3.1.1 In vitro studies

In 2007, Mahoney et al. encapsulated rat embryonic NPCs into a photopolymerized PEG-PLA (poly[lactic acid]) hydrogel using Darocur 2959 initiator and modified it with collagen and bFGF-2 [152]. PLA was introduced into the hydrogel and provided its degradability. It was found that PEG-PLA hydrogel supported intense cell proliferation without collagen and it was further increased with incorporated bFGF-2. In contrast, collagen did not increase cell viability, causing aggregation of NPCs and inhibiting the effects of bFGF-2.

Hynes et al. modified the PLL hydrogel with either a linear or a four-arm acrylated PEG photopolymerized using Irgacure 2959 photoinitiator [153]. Linear PEG-modified polymer remained undegraded after 24-hour exposure to trypsin while four-arm PEG modifications resulted in a higher stiffness. Incorporated mouse postnatal NPCs showed minimal degree of apoptosis after the photopolymerization. Four-arm PEG gel supported viability and promoted mostly neuronal differentiation of the cells by an unknown mechanism. The same group developed a library of 52 hydrogels composed of linear or four-arm PEG and PLL with varying molecular weights [154]. The hydrogels with four-arm PEG compositions contained a greater amount of free amines and were enzymatically degradable. With the mouse postnatal NSCs the high free-amine variants (> 3.0 μmoles/mg) were found to be cytotoxic. Hydrogels enhancing cell migration had elastic moduli ranging from 3.5 kPa to 5.5 kPa. Higher elastic moduli, for example with the “D3b” (70-150 kDa PLL cross-linked with 2 kDa PEG, NH_3_:OH ratio of 3:1) gel having a stiffness of 20 kPa led to decreased neuronal differentiation. Neuronal differentiation was more pronounced with the 70-150 kDa PLL hydrogels than with 150-300 kDa PLL hydrogels.

In a PNS study by Dadsetan et al., an oligo-(polyethylene glycol) fumarate was photocross-linked (Irgacure 2959 as photoinitiator) with [2-(methacryloyloxy)ethyl]-trimethylammonium chloride, which allowed for the fabrication of a cationic hydrogel [155]. The authors investigated rat embryonic DRG cells seeded on such hydrogels and found that it promoted neurite extension.

In 2009, Namba et al. developed a porous PEG-fibrin modified hydrogel network [156] and tested it with rat primary embryonic neural cells. The fibrin network was sequentially cleaved by collagenase but with no effect on cell viability. Such porous PEG scaffold enabled neurite outgrowth in contrast to non-porous and fibrin-containing hydrogels, which inhibited it.

The compatibility of the photocross-linking process with cells is still debatable. In a study by Mooney et al. (see also Subsection 2.7.) used photopolymerized PEG-PLA as a negative control for evaluation of photoencapsulation-induced apoptosis in fetal rat primary neuron cultures [120]. Darocur 2959 was used as the photoinitiator that cross-linked PEG via end-capping methacrylate groups. The photoencapsulation process resulted in massive cell death and increase of Caspase activity compared to fibrin matrix.

In 2010, Scott et al. developed two-component hydrogels composed of PEG and collagen type I and investigated their effects on surface-seeded DRG and PC12 cells [157]. The PEG-collagen conjugate was fabricated by covalent binding of acryl-PEG-N-hydroxysuccinimidyl to the amino groups of collagen. Next, the conjugate was mixed with PEG diacrylate in different proportions and cross-linked with Irgacure 2959 photoinitiator. The increase in PEG-collagen ratio reduced hydrogel stiffness and increased the mesh size, as the collagen terminated the growing PEG chain. The neurite extension of PC12 and embryonic chick DRG cells was markedly more improved in the softest 3% PEG gel with the highest collagen content.

In order to tune biodegradability, Lampe et al. photo-co-polymerized slowly degrading PEG with quickly-degrading PLA-b-PEG-b-PLA [158] via grafted methacrylate groups and Irgacure 2959 photoinitiator. The team investigated the effects of the resulting hydrogel on the encapsulated rat primary embryonic neurons: modification with PLA allowed for hydrogel degradation, while unmodified PEG induced cell death, oxidative stress, and slower proliferation.

The same team investigated the impact of PEG macromere concentration on photo-encapsulated rat embryonic NPCs and found that astro-glial differentiation and “glial-scar” gene expression increased with increasing PEG content [159]. Higher PEG content also increased apoptosis and reduced metabolic activity in cells. PEG with 7.5% (wt) had stiffness close to that of the native brain and was the only hydrogel that supported cell proliferation.

In 2011, Marquardt and Willits employed laminin binding to reduce the stiffness of the PEG hydrogel in order to study the growth of chick embryonic DRG photo-encapsulated into PEG diacrylate [160]. Laminin conjugation to acrylate groups blocked the cross-linking and decreased stiffness of the gel, while the increase in PEG increased the hydrogel stiffness. DRG cells displayed longer neurites in softer gels and gels modified with laminin.

In a PNS study, Curley and Moore utilized a two-component hydrogel system composed of PEG hydrogel without cells and RADA16-I hydrogel with suspension of rat embryonic DRG cells [161]. PEG hydrogel was photopolymerized via grafted methacrylate groups using Irgacure 2959 photoinitiator and its external layers were used as a support for RADA16-I in such ‘2.5D’ model. DRG neurite extension was observed both in RADA16-I and on the surface of PEG hydrogels, however neurites did not grow into the PEG layer, which also tended to detach.

Tamariz et al. used a PEG-Si (a thixotropic hydrogel with dispersed silica nanoparticles) hydrogel to create a functional gradient of recombinant semaphorin 3A to enhance axonal outgrowth from rat embryonic dopaminergic neurons [162]. In this study, a PEG-Si drop that contained mouse IgG-Alexa Fluor 488 conjugate was embedded in collagen to simulate interaction with the extracellular matrix *in vitro*. PEG-Si provided gradual diffusion of IgG-Alexa Fluor 488 conjugate into the adjacent collagen matrix. The Semaphorin 3A-containing system increased axonal growth in a dopaminergic culture. PEG-Si showed no cytotoxicity for neurons and biodegraded in a simulated body fluid.

A similar approach was employed by Lee et al. to develop a thixotropic system comprised of photocross-linked PEG hydrogel with embedded PLGA microparticles loaded with IGF-1 (insulin-like growth factor 1) [163]. Such a system was expected to produce a propagating gradient of the drug for axonal growth direction. In addition, authors modified the hydrogel surface with the axon-guiding fibronectin and laminin and tested it with suspension of mouse ES-NPC. The axons reached the targeted length (5 mm) within 10 days with neuroblasts migrating along the axons.

#### 3.1.2 In vivo studies

In 2008, Bjugstad et al. implanted a PEG-based hydrogel into primate striatum and frontal cortex in a green monkey model [164]. The PLA-b-PEG-b-PLA triblock polymer was photocross-linked via grafted methacrylate groups (Irgacure 2959 as photoinitiator) to form a hydrogel. One hemisphere received a hydrogel injection, the contralateral hemisphere received no injection but was exposed to needle damage similar to what occurs during the injection procedure (sham-implanted group). One animal also received a bilateral PEG-GDNF injection. After 4 months, all hydrogels completely degraded. A softer 13% w/v PEG hydrogel caused minimal astro- and microglial infiltration similar to that in sham-treated group, while 20% w/v PEG and GDNF produced only a slight increase in glial response. Thus, GDNF remained active demonstrating that PEG was a promising drug delivery system. In 2010, the same team implanted a similar hydrogel into the rat brain close to *substantia nigra* [165]. The hydrogel showed insignificant swelling *in vivo* but did not degrade completely over the course of 56 days. All hydrogels (fast-degrading, slow-degrading, and non-degrading) caused significantly weaker astrogliosis when compared to the sham group. Fast-degrading hydrogels caused the weakest microglial response even when compared to the sham group.

In the *in vivo* part of the study by Tamariz et al., PEG-Si was injected into the rat striatum [162]. The contralateral hemisphere received an injection of sterile saline. A significant increase in inflammatory and astroglial responses was observed after 30 days in the polymer-injected hemispheres.

In 2011, Lampe et al. injected into the caudal area of the rat brain near the *substantia nigra* and the rostral area adjacent to the striatum with PLA-b-PEG-b-PLA triblock-derived hydrogel with embedded PLGA microparticles and loaded with GDNF and BDNF [166]. Both hydrogel systems provoked weaker inflammatory responses than with control BSA injection and initial swelling was minimal. BDNF-bearing hydrogel induced astrocytic invasion.

In conclusion, PEG-based hydrogels possess poor physical properties and are intrinsically non-interactive until they are modified. Collagen and laminin modifications of PEG can reduce the hydrogel stiffness, while polylactide modification helps its biodegradability. The majority of methods of cell encapsulation into PEG hydrogel are based on photo/chemical cross-linking, potentially affecting their viability. *In vivo* studies revealed inflammatory reactions following PEG hydrogel implantation. Thus, PEG hydrogel systems seem to be inferior compared to the natural hydrogels, for brain tissue regeneration. Overall comments on PEG-based hydrogels properties for brain tissue regeneration are provided in Supplementary table S10.

### 3.2 Methacrylates and methacrylamides

Methacrylate is extensively used for fabrication of PEG-based hydrogels. We focus here on mono-component methacrylate- (MA-) and methacrylamide- (MAA-) based hydrogels, mostly used in *in vivo* studies of SCI repair [12,[15], [16], [17],^19^,^151^].

#### 3.2.1 In vitro studies

In 2008, Woerly et al. modified a pHPMA (poly[N-(2-hydroxypropyl) methacrylamide]) hydrogel with sialic acid, which can be recognized by growth factor receptors [167]. The acrylated 3′-sialyllactose was copolymerized with HPMA monomers via free radical cross-linking co-polymerization. Sialyllactosyl did not significantly influence porosity of the hydrogel, but did reduce its swelling.

In a PNS study by Jhaveri et al., pHEMA (poly[2-hydroxyethyl methacrylate]) was tested as a neurotrophin delivery system in rat postnatal DRG culture [168]. In order to control the NGF release rate, the hydrogel was modified in two variants, namely pHEMA-Lys and pHEMA-NaCl: covalent binding of lysine provided the hydrogel with positive charge and reduced NGF release. The preparation of the hydrogel in 0.6 M NaCl solution enlarged the pores and induced the NGF release. However, the hydrogels remained poorly porous and extremely stiff. Elastic moduli were 93 kPa for pHEMA-NaCl hydrogel and 350 kPa for pHEMA-Lys hydrogel, which was softer than the unmodified variant. Significantly longer neurites were grown from DRG explants when NGF was released from pHEMA-Lys hydrogel; this was likely due to longer exposure to the drug.

In 2010, Kubinova et al. published a study on human fetal NSC behavior in IKVAV-peptide-modified p(HEMA-AEMA) superporous hydrogel [169]. The authors revealed the need for grafting adhesive moiety to the hydrogel to achieve acceptable cell distribution, differentiation, and maturation.

In 2014, Pertici et al. used a PLA-b-pHEMA hydrogel in a SCI model [170]. The *in vitro* part of the study confirmed that the hydrogel was degradable and porous. Embryonic rat spinal motor neurons showed robust neurite growth proving lack of cytotoxicity for the hydrogel.

#### 3.2.2 In vivo studies

Woerly et al. injected Parkinson’s disease model rat striatum with cell-free sialic acid-functionalized pHPMA hydrogel [167]. The hydrogels remained stable after 4 months of observation, integrated well with host parenchyma and induced only small inflammatory response with no glial scars formation. The hydrogels also stimulated vascularization, host cell migration and differentiation towards neurons and stimulated growth of dopaminergic nerve fibers.

In 2008, Hejcl et al. implanted rats with pHEMA hydrogel to the site of spinal cord transection [171]. The hydrogel integrated well with the tissue, ameliorated the glial response and stimulated axonal in- and trans-growth. In another study, the same group implanted the balloon-induced compression lesion with either blank pHPMA-RGD or the hydrogel populated with rat MSCs during shaking [172]. In such chronic spinal cord injury model, the hydrogel stimulated axonal growth, neuronal migration, but did not prevent formation of astro-glial scars. However, marked functional improvement was noted in animals treated with the hydrogel-MSC system.

In 2013, Kubinova et al. utilized highly porous poly-[2-hydroxyethyl methacrylate-*co*-2-aminoethyl methacrylate] (p[HEMA-AEMA]) hydrogels and their modifications with SIKVAV peptide to implant into hemi- or trans-sectioned spinal cord of rats [173]. From the types of hydrogel tested, the variant with moderate porosity (68%) and moderate stiffness (27 kPa) showed the best axonal ingrowth stimulation and gave no rise in cavity formation.

Another SCI study was published by Pertici et al. [174]. This group injected the site of spinal cord injury with blank pHPMA hydrogel. The hydrogel successfully bridged the defective spinal cord leading to better functionality when compared to the untreated group. The hydrogel promoted axonal growth but led to an increase in macrophage-monocyte infiltration. However, the process of axonal regeneration can also be guided by microglia. The authors speculated that such inflammatory infiltration was due to the cell retention within the gel.

Li et al. described the effects of pHEMA behavior in rat microscale spinal cord lesion model [175]. They confirmed that pHEMA attenuated astro-glial response and production of neuro-inhibiting neurocan (CSPG) but at the same time recruited microglia cells. The scaffold was pushed out from the lesion site, which indicated it as being unsuitable for micro lesion treatment.

In the *in vivo* part of the SCI study by Pertici et al., the PLA-b-pHEMA hydrogel was implanted into rat spinal cord at hemitransection injury site [170]. The results indicated that the hydrogel induced axonal regeneration and prevented glial scar formation, however, microglia and macrophage infiltration occurred eight weeks post-surgery.

In conclusion, the main drawbacks for MA- and MAA-based hydrogels are non-biodegradability and high stiffness independent of existing modifications. These disadvantages make them generally unsuitable for implantation into the brain that has been mirrored in the *in vivo* studies. However, these materials might be promising for spinal cord injury repair. Overall comments on MA- and MAA-based hydrogels in brain tissue regeneration are given in Supplementary table S11.

## 4 Summary and outlook

The role of hydrogels in nervous system regeneration in general and in spinal cord regeneration in particular has been widely reviewed [[19], [20], [21], [22],^28^,^70^,^105^]. However, the structural and chemical properties of usable hydrogels have not been sufficiently studied [133,176,177]. In this review, we performed analysis of the recent literature data related to the applicability of hydrogels for brain tissue regeneration with the focus on essential parameters of each hydrogel type, and their advantages and disadvantages. Table 1 briefly summarizes data from the reviewed articles.

**Table 1:** Summary of the hydrogel types and their applicability for brain tissue regeneration.

Hydrogel	Origin	Immuno-genicity	Bio-degradability	Porosity	Bio-compatibility	Neuronal differentiation	Neurite outgrowth
HA	Natural	−	++	+	+	+	+
Collagen type I	Natural	±	+	+	+	+	+
Alginate	Natural	−	−	−	+	+	+
Chitosan	Natural	−	+	+	+	+	−
(HA)methyl-cellulose	Natural	−	+	+	+	+	+
Matrigel	Natural	+	+	+	±	+	+
Fibrin	Natural	±	+	+	+	+	+
Gellan gum	Natural	±	+	+	±	−	±
Self-assembling peptides	Semi-natural	±	+	+	+	+	+
PEG	Synthetic	−	−	±	+	−	+
MA/MAA	Synthetic	+	−	+	+	+	+

Abbreviations: *+:* suitable; −: unsuitable; ±: need further studies. HA- hyaluronic acid; PEG – poly(ethyleneglycol); MA – methacrylate; MAA – methacrylamide.

Our analysis arranges hydrogels for brain injury repair into three principal categories: (1) potentially applicable for brain injury therapy (HA-, collagen type I-, alginate-, chitosan-, methylcellulose-, fibrin-, and self-assembling peptide-based hydrogels), (2) hydrogels that do not meet basic requirements for human brain injury therapy (potentially carcinogenic Matrigel and stiff and non-degradable MA- and MAA-based synthetic materials), and (3) hydrogels that hold promise for further investigations (gellan gum-, PEG-based, and other multi-component systems).

It is clear that the ‘plain’ hydrogel matrices are rarely sufficient for brain tissue engineering. Even modified hydrogels impregnated with cells are not deprived of significant drawbacks like insufficiency of neurite guidance, poor integration, and probability of side effects and other clinical complications such as formation of the epileptic focus. Thus, the future design of hydrogels must target the production of complex hybrid systems involving the neuroprosthetic [178,179] and bioprinting [6] technologies, exploring other biocompatible and naturally occurring polymers and possibly developing new *in vitro* and *ex vivo* models of neuroregeneration.

## Conflict of interest statement

We declare that we have no conflict of interest.

## Acknowledgements

The work was supported by the Russian Science Foundation (RSF) grant No. 17-15-01487 (1, 2), the Russian academic excellence project ‘5-100’ (Section3), and by the Science Foundation Ireland (SFI) grant 13/SIRG/2144 (Section4). We are grateful to T. Foley (Department of Anatomy and Neuroscience, UCC) for critical reading of the manuscript.

## Appendix A Supplementary data

Supplementary material Image 1
