# Supplementary material for: Hydrogel-assisted neuroregeneration approaches towards brain injury therapy: A state-of-the-art review
Source: Comput Struct Biotechnol J. 2018 Nov 2;16:488–502. doi: 10.1016/j.csbj.2018.10.011 (PMC6232648; doi:10.1016/j.csbj.2018.10.011)
Supplement: Supplementary file 1 — Supplementary material [file mmc1.pdf]

Appendix A. Supplementary data

Table S1. Inherent characteristics of hyaluronic acid (HA) as a candidate for hydrogel-based brain tissue regeneration.

Positive findings	Negative findings
• natural component of the CNS (biocompatible) [23,25,41,44,46]<br>• immunoneutral [32,65]<br>• biodegradable [32]<br>• highly porous [34,44]<br>• stimulates neuronal viability and differentiation of NS/PCs, NPCs, and iPS-NPCs *in vitro* [32,41,42,49]<br>• stimulates neurite outgrowth of HNPCs *in vitro* [40]<br>• promotes neuronal differentiation of hiPS-NPCs *in vivo* [14,47]<br>• holds antigliosis and anti-inflammatory effects *in vivo* [45,46]<br>• supports survival and proliferation of C17.2 and ReNcell *in vivo* [65]<br>• shields transplanted cells from host immune response *in vivo* [14,65]<br>• HA-PLGA co-gel provides functional improvement *in vivo* [46]<br>• does not influence gel stiffness in co-gels [55]<br>• superior to Matrigel in terms of neurite outgrowth in SH-SY5Y cell line *in vitro* [38]<br>• HA-modified alginate supports greater cell viability and neuronal differentiation of ESCs and NSCs and neurite extension when compared to unmodified alginate [78]<br>• HA is a shear-thinning agent in co-gels [96]	• poor adhesiveness [32,34,36,49]<br>• does not interact with integrins [23,35]<br>• does not support hiPS-NPCs viability *in vivo* [14]<br>• requires modifications to reduce the biodegradation rate [23,34,35,49]<br>• requires another component (e.g. collagen) for stabilization [49]<br>• swelling [93]

NS/PCs – neural stem/progenitor cells – a heterogeneous cell population; NPCs – neural progenitor cells; iPS-NPCs – induced pluripotent stem cell-derived neural progenitor cells; hiPS-NPCs – human induced pluripotent stem cell-derived neural progenitor cells; C17.2 – a mouse immortalized NSC line; ReNcell – human immortalized NPC line; PLGA – poly(lactic-co-glycolic acid); SH-SY5Y – human neuroblastoma cell line; ESCs – embryonic stem cells; NSCs – neural stem cells

Table S2. Inherent characteristics of collagen type I as a candidate for hydrogel-based brain tissue regeneration

Positive findings	Negative findings
• biocompatible [48,62–64]<br>• non-toxic[64]<br>• biodegradable [48]<br>• non-immunogenic [21,63–65] (though negated by Itosaka *et al.* [113])<br>• self-healing *in situ* [64]<br>• reduces micro- and astrogliosis *in vivo* [64,66]<br>• gels under physiological conditions [49]<br>• supports differentiation of BMSCs towards neuronal lineage [59]<br>• retains and stabilizes HA [49,63]<br>• supports viability, proliferation, and differentiation of embryonic, postnatal, and adult NS/PCs when mixed with HA [49,63]<br>• supports neurite outgrowth [53,58,60,157]	• does not naturally occur in mammalian brain tissue, except subventricular zone [49,61]<br>• has no measurable effect on NSC's viability [57,152]<br>• decreases in volume post gelation both *in vitro* and *in vivo* [64]<br>• increases stiffness in co-gels [55,59]<br>• inferior to Matrigel and self-assembling peptides in terms of NSCs survival and differentiation [61]<br>• inferior to Matrigel in terms of neural differentiation and neurite growth in hESCs experiments *in vitro* [107]<br>• inferior to Matrigel in terms of NSCs viability, differentiation, and migration *in vitro* [61]

BMSCs – bone marrow-derived stem cells; hESCs – human embryonic stem cells

Table S3. Inherent characteristics of alginate as a candidate for hydrogel-based brain tissue regeneration

Positive findings	Negative findings
• non-toxic [68,71–73,78,91]<br>• biocompatible [78]<br>• non-inflammatory [78]<br>• shields NSCs from host immune response [78]<br>• neuro-protective [73,75]<br>• stimulates structural and functional maturation of NSCs, glial cells [74,75]<br>• supports NSCs viability and proliferation [71,74,78]<br>• promotes self-healing when used as a cross-linker for the chitosan-based hydrogel [91]<br>• chitosan-alginate co-gel supports viability and neuronal differentiation of NSCs [91]<br>• alginate-poly(γ-glutamic acid) ICC scaffolds are highly porous, non-cytotoxic to iPS-NPCs and promote their neuronal differentiation [76]	• does not naturally occur in mammalian brain tissue [67,68]<br>• poorly recovers after shear stress [77]<br>• hardly injectable [77]<br>• non-degradable [68]<br>• degradation products decrease NPCs proliferation [68]<br>• insufficient porosity [68,74]<br>• requires modifications to provide cell attachment and survival [74,78]<br>• supports worse NSCs spheroids proliferation and differentiation when compared to chitosan-based hydrogel [77]<br>• requires cross-linking to prevent diffusion *in vivo* [78]

Table S4. Inherent characteristics of chitosan as a candidate for hydrogel-based brain tissue regeneration

Positive findings	Negative findings
• thermally cross-linkable [84]<br>• highly porous [85,86,90]<br>• biodegradable [85]<br>• non-immunogenic [83]<br>• non-toxic to NSCs [77,91]<br>• cell-adhesive for cervical ganglion neurons and embryonic cortical neurons [82,85]<br>• decreases stiffness in chitosan-agarose co-gel [82]<br>• suits for drug delivery systems [89]<br>• favors NSC spheroids' proliferation and differentiation [77]<br>• improves functional recovery in zebrafish embryos when injected with neurosphere NSCs [77]<br>• chitosan-alginate co-gel supports viability and neuronal differentiation of NSCs [91]	• long gelling time [84,89]<br>• can induce cationic cytotoxicity [90]<br>• requires modifications to support cell viability and neurite outgrowth [84,85]<br>• requires modifications for self-healing [77,89,91]

Table S5. Inherent characteristics of methylcellulose- and HAMC- as candidates for hydrogel-based tissue regeneration

Positive findings	Negative findings
• gelling at physiological temperatures [15]<br>• injectable [15,93,94,97,101,103]<br>• biodegradable [103]<br>• exhibit anti-inflammatory [93,97–100,103] and anti-astrogliosis effects [98–100,103]<br>• non-toxic [97,103]<br>• applicable to drug delivery systems [92,93,95–100]<br>• support viability of NS/PCs [94]<br>• improve the solubility of sparingly soluble drugs [92]<br>• methylcellulose reduces swelling in co-gels [93,94,103]<br>• methylcellulose is self-healing [96,103]<br>• HAMC prevents cell sedimentation and aggregation [94,102]<br>• HAMC promotes NSC viability and penetration into host brain *in vivo* [102]	• methylcellulose exhibits low hydrophilicity unless blended with HA or modified [92]<br>• barely functionalizable backbone [96]

Table S6. Inherent characteristics of Matrigel as a candidate for hydrogel-based brain tissue regeneration

**Positive findings**	**Negative findings**
• contains growth factors and adhesive proteins [15,20,22,26,104]<br>• injectable [15]<br>• porous [180]<br>• physiological stiffness [106]<br>• growth factor-reduced form exhibits an anti-inflammatory effect, supports ES-NPCs viability and neuronal differentiation, and promotes host cell proliferation *in vivo* [109]<br>• promotes NS/PCs maturation and functionality in MCAO model [112]<br>• superior to collagen I in terms of neuronal differentiation and neurite growth in hESCs experiments *in vitro* [107]<br>• growth factor reduced form is superior to RADA16-I in terms of viability, migration, and maturation of ES-NPCs *in vitro* [109]<br>• superior to collagen I in terms of NSCs viability, neuronal differentiation, and migration *in vitro* [61]<br>• supports viability and maturation of spiral ganglion neurons *in vitro* [111]<br>• applicable for modification of porous PEG scaffold to promote rat neurons' neurite outgrowth [156]	• unstandardized composition [15,66,105,109]<br>• immunogenic [15]<br>• solidifies at room temperature [108]<br>• inferior to salmon fibrin in terms of stimulating neurite growth of cortical and spinal neurons *in vitro* [106]<br>• inferior to RADA16-I in terms of NSCs survival and neuronal differentiation *in vitro* [108]<br>• inferior to RADA16-I hydrogel in terms of NSCs viability *in vitro* [61]<br>• inferior to HA in terms of neurite outgrowth in SH-SY5Y cell line *in vitro* [38]<br>• increases stiffness in co-gels [110]

ES-NPCs – embryonic stem cell-derived neural progenitor cells; MCAO – middle cerebral artery occlusion; RADA16-I – a 16-mer peptide consisting of four RADA (arginine, alanine, aspartate, alanine) tetramers, also known as PuraMatrix; PEG – poly(ethylene glycol)

Additional citation:

[180] Balachandran NTL and HL and NMS and K. Fabrication of a matrigel–collagen semi-interpenetrating scaffold for use in dynamic valve interstitial cell culture. Biomed Mater 2017;12:45013.

Table S7. Inherent characteristics of fibrin as a candidate for hydrogel-based brain tissue regeneration

Positive findings	Negative findings
• easily tunable mechanical properties [113,116,120,121]<br>• porous [121]<br>• biodegradable [15,119,121]<br>• injectable [15,113,122]<br>• bioactive (contains RGD peptide) [119,121]<br>• non-toxic [120]<br>• can be produced from patient's own blood (non-immunogenic) [113]<br>• advantageous drug delivery platform [116–118,122]<br>• promotes neuronal differentiation and maturation rather than glial differentiation of NSCs *in vitro* [120]<br>• supports viability and promotes neuronal differentiation and migration of BMSCs *in vivo* [113]<br>• salmon fibrin is superior to Matrigel in terms of stimulating neurite growth of cortical and spinal neurons *in vitro* [106]<br>• Tisseel fibrin gel is superior to PEG in terms of NPC neuronal differentiation *in vitro* [159]	• pro-inflammatory [114,115]<br>• requires modifications for proper neuron-glial differentiation of ES-NPCs *in vitro* [118]<br>• inferior to collagen type I in terms of DRG neurite outgrowth stimulation *in vitro* [53]

RGD – a cell-adhesive tripeptide (arginine-glycine-aspartate); DRG – dorsal root ganglion

Table S8. Inherent characteristics of gellan gum as a candidate for hydrogel-based brain tissue regeneration

Positive findings	Negative findings
• non-toxic [16,129]<br>• resistant to acid stress [16]<br>• injectable [124,126]<br>• *in situ* gelling [124]<br>• porous [127,129]	• promotes oligodendrocytal differentiation of NS/PCs *in vitro* [126]<br>• aggregates NS/PCs [126]<br>• requires purification from divalent cations prior to injection [127,128]<br>• requires modifications to support cell viability, differentiation and neurite outgrowth [127–129]

Table S9. Inherent characteristics of self-assembling peptides and proteins as candidates for hydrogel-based brain tissue regeneration

Positive findings	Negative findings
• biocompatible at working concentration [61,137,143]<br>• biodegradable [130,149]<br>• non-immunogenic [61]<br>• RADA16-I is porous [137,139,143]<br>• RADA16-I is injectable and self-healing [104,148,149]<br>• RADA16-I supports NSC migration and differentiation into neurons and astrocytes *in vitro* [108]<br>• Keratin-based hydrogels support NS/PC survival *in vitro* [136]<br>• RADA16-I is an advantageous and easy-tunable drug-delivery system *in vitro* [137,138]<br>• RADA16-I supports NSC viability *in vitro* [143] and *in vivo* [149]<br>• RADA16-I is mechanically tunable [143]<br>• RADA16-I improves viability, differentiation, morphological and functional maturation of ES-NPCs [129]<br>• keratin-based hydrogels are highly porous [147]<br>• keratins contain internal RGD motifs [147]<br>• K_xL_y, R_xL_y and E_xL_y are easily injectable, porous, cause minimal gliosis and inflammation, exhibit no evident toxicity to neurons and induce vascularization *in vivo* [130]<br>• RADA16-I readily integrates with host tissue and reduces micro- and astrogliosis *in vivo* [104,148]<br>• RADA16-I is superior to Matrigel in terms of NSC survival and differentiation *in vitro* [108] (though neglected by [109])<br>• RADA16-I is superior to Matrigel in terms of NSC viability *in vitro* [61]<br>• elastin-like proteins support DRG viability *in vitro* [146]<br>• human keratin promotes neurite growth and vascularization of peripheral nerves *in vivo* [147]<br>• RADA16-I promotes neurite outgrowth and supports survival of iPS-NPCs *in vivo* [104]	• RADA16-I is toxic to human NSCs at concentrations above 1% [108]<br>• keratins are slowly degradable [147]<br>• K_xL_y, R_xL_y and E_xL_y support limited ingrowth of nerve fibers and neuron-supportive astroglia [130]<br>• RADA16-I does not promote migration of neurons and oligodendrocytes and does not prevent host cells from apoptosis [148]<br>• RADA16-I is inferior to growth factor-reduced Matrigel in terms of viability, migration, and maturation of ES-NPCs *in vitro* [109]

K_xL_y, R_xL_y, and E_xL_y – diblock copolypeptide hydrogels including combinations of lysine and leucine (K_xL_y), arginine and leucine (R_xL_y), and glutamate and leucine (E_xL_y)

Table S10. Inherent characteristics of PEG as a candidate for hydrogel-based brain tissue regeneration

Positive findings	Negative findings
• highly hydrophilic [158]<br>• supports neurite extension at low concentrations (PC12 and DRG cells) *in vitro* [157,160]<br>• 4-arm PEG-cross-linked PLL hydrogels are biodegradable and promote viability, proliferation, and neuronal differentiation of NPCs and NSCs *in vitro* [153,154]<br>• PEG-RADA16-I composite supports DRG neurite outgrowth *in vitro* [161]<br>• IGF-1 gradient in PEG-PLGA system directs axonal growth *in vitro* [163]<br>• PLA-b-PEG-b-PLA-based hydrogels attenuate glial response *in vivo* [165,166]<br>• PEG-Si is thixotropic (shear-thinning and self-healing) [162]<br>• a PLA-b-PEG-b-PLA triblock-derived hydrogel is biocompatible and minimally-swelling [166]	• poorly porous [156,159]<br>• non-degradable [152,157–159,162,164,165]<br>• bioinert [160]<br>• photo-encapsulation of primary neurons induces apoptosis [120]<br>• poorly supports neuronal differentiation of NPCs *in vitro* [159]<br>• requires modifications to support cell viability and proliferation [152,158]<br>• PEG-Si increases glial response *in vivo* [162]

PC12 – rat pheochromocytoma cells; PLL – poly-L-lysine; IGF-1 – insulin-like growth factor 1; PLA-b-PEG-b-PLA – triblock polymer built of poly(lactic acid) and poly(ethylene glycol);PEG-Si – a thixotropic PEG-based hydrogel with dispersed silica nanoparticles

Table S11. Inherent characteristics of MA- and MAA-based polymers as candidates for hydrogel-based tissue regeneration

Positive findings	Negative findings
• pHPMA is highly porous [167]<br>• pHEMA can be modified to tune drug release kinetics *in vitro* [168]<br>• sialic acid-modified pHPMA is highly biocompatible, stimulates vascularization, host cell migration, their neuronal differentiation, TH-positive fiber growth, and prevents gliosis *in vivo* [167]<br>• pHEMA integrates with host spinal cord, stimulates neurofilament growth and attenuates glial response *in vivo* [171]<br>• pHPMA-RGD stimulates axonal growth and neuronal migration *in vivo* [172,174]<br>• pHEMA attenuates astrogliotic response and inhibits the synthesis of neuroinhibitory CSPG *in vivo* [175]<br>• PLA-b-pHEMA is non-toxic to spinal motoneurons and allows neurite outgrowth *in vitro* [170]<br>• PLA-b-pHEMA stimulates axonal growth and prevents glial scar formation *in vivo* [170]	• pHEMA and pHPMA are non-degradable [12,16,167,170,175]<br>• pHEMA is stiff [168]<br>• pHPMA is swelling [167]<br>• pHPMA does not prevent glial scarring *in vivo* [172]<br>• pHPMA and pHEMA induce microglial infiltration *in vivo* [174,175]<br>• PLA-b-pHEMA induces microglial and macrophageal response *in vivo* [170]

pHPMA – poly(N-[2-hydroxypropyl] methacrylamide); pHEMA – poly(2-hydroxyethyl methacrylate); PLA-b-pHEMA – a block copolymer hydrogel built of poly(lactic acid) and poly(2-hydroxyethyl methacrylate); TH – tyrosine hydroxylase (the key dopamine synthesis enzyme)
